# Editorial: Multidimensional development of student-athletes: new perspectives on dual career

**DOI:** 10.3389/fspor.2025.1701681

**Published:** 2025-09-19

**Authors:** Ricardo T. Quinaud, Mojca Doupona, Flavia Guidotti, Laura Capranica

**Affiliations:** ^1^ School of Sport, University of Extreme South of Santa Catarina, Criciúma, Santa Catarina, Brazil; ^2^ European Athlete as Student Network, Ghaxaq, Malta; ^3^ Department of Sport Sociology and History, Faculty of Sports, University of Ljubljana, Ljubljana, Slovenia; ^4^ Department of Human Sciences and Promotion of the Quality of Life, San Raffaele Roma Open University, Rome, Italy; ^5^ Department of Movement, Human and Health Sciences, University of Rome “Foro Italico”, Rome, Italy

**Keywords:** athlete, student, athletes' career, career support, sport, education

**Editorial on the Research Topic**
Multidimensional development of student-athletes: new perspectives on dual career

## Introduction

The phenomenon of dual careers in sports, in which sportspersons (e.g., athletes, coaches, physical trainers, referees, sports managers, and volunteers) simultaneously pursue sporting excellence and academic/professional development, has been the focus of growing attention from scholars and policymakers. For decades, dual career was often studied from segmented or unidimensional perspectives, emphasizing either sport or education. In addressing dual career, it is crucial to acknowledge that this concept extends beyond academic education, encompassing also vocational training as a key dimension in the development of student-athletes.

A substantial body of literature has emphasized the importance of holistic development systems, calling for the integration of efforts from multiple stakeholders to effectively sustain athletes throughout their educational and sporting pathways. In parallel, psychological research has made significant contributions to the dual career field, deepening our understanding of the personal, social, and emotional dynamics involved in balancing sport and education. Moreover, the area of career transitions has emerged as a relevant niche, highlighting the complexities athletes face when navigating changes within and beyond sport.

By incorporating these perspectives, the relevance of our special issue is further reinforced, as it seeks to address critical gaps in literature and practice while advancing the view of dual career as a multidimensional and evolving process. The challenges dual career sportspersons face are inherently multidimensional, shaped by the interplay of institutional frameworks, cultural contexts, psychosocial resources, health, and equity. The 14 contributions included in this Special Issue of Frontiers in Sports and Active Living advance this field by offering a comprehensive understanding of dual careers and highlighting the urgent need for integrative, evidence-based solutions.

### Institutional policies and structural conditions

Dual careers cannot be understood without considering the structural conditions under which they unfold. Multiple studies in this collection demonstrate that institutional and systemic policies decisively shape opportunities for dual career sportspersons. Literature reviews have shown that while progress has been made across Europe, barriers remain in areas such as financial support, access to specialized services, and coordination between educational and sporting institutions. For example, although Brazilian universities have advanced in offering holistic support, limitations persist in financial aid and career transition strategies da Silva. Similarly, in the Republic of Kosovo, insufficient awareness and a lack of integrated frameworks hinder the effective support of student-athletes Gjaka et al.

At the European level, divergences in perception between student-athletes and higher education experts were found, highlighting the need for mutual understanding and more transparent policy development. Complementing these findings, research mapping 31 institutional benefits across Europe underscored the importance of academic flexibility, justified absences, and access to sports facilities; however, it revealed wide variations in implementation. A specific example from Italy further showed that the introduction of dual career regulations at the University of Rome “Tor Vergata” led to greater participation in both academics and sports, illustrating how institutional commitments can yield tangible results Cariati et al.

### Cultural contexts and comparative insights

Beyond institutional structures, cultural contexts exert a significant influence on how dual careers are experienced. In Spain, research revealed that identity, academic specialization, and competitive level strongly shape the choices and trajectories of student-athletes. Broader comparative studies between European and Brazilian contexts demonstrated that motivation and identity cannot be explained solely through individual traits but are deeply conditioned by social and organizational factors Gonçalves et al.

### Identity, employability, and long-term transitions

The experience of dual career sportspersons extends beyond their current performance. It is intimately tied to identity formation and employability. An Italian study on employability highlighted how personal attributes, human capital, and social capital work together to influence transitions beyond sports. Similarly, findings from comparative research have shown that motivation and identity are mediated by both institutional support and cultural expectations, affecting how student-athletes envision their careers after sports. Additionally, the Talent Project validated standards for the identification of student-athlete talent and guidelines for dual career support. Integrating these standards into institutional procedures, schools and clubs can align academic and sports requirements from the early stages of education, strengthening the school–club–family alliance and fostering more sustainable academic–sports transitions Vicari et al.

### Equity, diversity, and inclusion

A multidimensional perspective cannot neglect questions of equity and inclusion. Gender inequality remains a central challenge, as shown by the experiences of Italian women's football players, who continue to face disparities in recognition, financial resources, and institutional support. These inequalities reinforce the dual burden faced by women athletes, who must balance underfunded sports environments with demanding academic responsibilities. In addition, cultural and family pressures can also constrain the lives of student-athletes. Evidence from the Republic of Korea revealed how the internalization of extreme filial piety can lead to negative manifestations such as burnout, dropout, or disengagement (“athlete melt”), which compromise both athletic and academic development Yoon and Lim.

### Mental health, well-being, and workload management

The sustainability of dual careers also depends on the ability of sportspersons to manage the psychological and physical demands associated with their multiple roles. Mental health literacy has emerged as a crucial protective factor: higher levels of literacy are associated with reduced symptoms of anxiety and depression, while gaps in knowledge perpetuate psychological distress Usenik and Kranjec. These findings align with global calls to integrate mental health education into sports development programs. Equally pressing is the question of workload balance. A longitudinal study of elite handball players highlighted how training intensity, combined with academic and psychosocial stressors, heightens vulnerability to injury and illness Drole et al.

### Toward multidimensional ecosystems of support

Overall, these findings: (I) emphasize that dual career success is closely linked to systemic factors. Fragmented or absent policies risk leaving sportspersons unsupported, while harmonized, context-sensitive approaches provide a stronger foundation for balancing educational and athletic demands; (II) caution against universal policy transfers and instead advocate for culturally sensitive approaches that respect national and regional particularities; (III) illustrate that fostering resilience, adaptability, and employability is as critical as supporting sports and academics, ensuring sportspersons to be prepared for multiple futures; (IV) remind us that equitable dual career systems must not only create generalized opportunities but also address the specific barriers shaped by gender, disability, and cultural expectations; (V) emphasize that dual career programmes cannot ignore the biopsychosocial dynamics of athlete health. Sustainable strategies must include flexible scheduling, adequate recovery time, and robust psychosocial support systems. Additionally, there is a need for further developing digital resources to help athletes managing their dual career path, which has been widely addressed through multiple European funded projects over the past decade.

Taken together, the contributions in this Special Issue underscore that dual careers are inherently multidimensional phenomena. Institutional frameworks, cultural contexts, psychosocial resources, employability strategies, equity concerns, and health considerations are deeply interwoven. Focusing on one dimension in isolation, be it academic concessions, financial aid, or mental health, cannot ensure sustainable outcomes. Instead, successful dual career systems must be built as ecosystems that integrate these multiple dimensions into coherent, sportsperson-centered support structures.

Moving forward, three priorities stand out. First, more longitudinal and mixed-methods research is needed to capture the evolving nature of dual career trajectories across different career stages. Second, diversity and equity must be systematically embedded in dual career programs to ensure inclusive opportunities for women, sportspersons with disabilities, and those in under-resourced contexts. Third, stronger cross-sector collaboration is necessary, linking universities, sports organizations, governments, and families to provide comprehensive and consistent support. By embracing this multidimensional approach, the field will not only address the current challenges but also prepare sportspersons for successful futures beyond sports.

